# Defining the Genomic Signature of Totipotency and Pluripotency during Early Human Development

**DOI:** 10.1371/journal.pone.0062135

**Published:** 2013-04-17

**Authors:** Amparo Galan, Patricia Diaz-Gimeno, Maria Eugenia Poo, Diana Valbuena, Eva Sanchez, Veronica Ruiz, Joaquin Dopazo, David Montaner, Ana Conesa, Carlos Simon

**Affiliations:** 1 Valencia Node of The National Stem Cell Bank, Centro de Investigación Príncipe Felipe (CIPF), Valencia, Spain; 2 Bioinformatics Department and Functional Genomics Node (INB), Centro de Investigación Príncipe Felipe (CIPF), Valencia, Spain; 3 Fundación IVI (FIVI)-Instituto Universitario IVI (IUIVI)-Universidad de Valencia, INCLIVA, Valencia, Spain; University of Georgia, United States of America

## Abstract

The genetic mechanisms governing human pre-implantation embryo development and the *in vitro* counterparts, human embryonic stem cells (hESCs), still remain incomplete. Previous global genome studies demonstrated that totipotent blastomeres from day-3 human embryos and pluripotent inner cell masses (ICMs) from blastocysts, display unique and differing transcriptomes. Nevertheless, comparative gene expression analysis has revealed that no significant differences exist between hESCs derived from blastomeres versus those obtained from ICMs, suggesting that pluripotent hESCs involve a new developmental progression. To understand early human stages evolution, we developed an undifferentiation network signature (UNS) and applied it to a differential gene expression profile between single blastomeres from day-3 embryos, ICMs and hESCs. This allowed us to establish a unique signature composed of highly interconnected genes characteristic of totipotency (61 genes), *in vivo* pluripotency (20 genes), and *in vitro* pluripotency (107 genes), and which are also proprietary according to functional analysis. This systems biology approach has led to an improved understanding of the molecular and signaling processes governing human pre-implantation embryo development, as well as enabling us to comprehend how hESCs might adapt to *in vitro* culture conditions.

## Introduction

Totipotency and pluripotency are at the root of both embryo development and the stem cell field. Therefore, understanding the molecular mechanisms involved is crucial to understanding developmental biology as well as regenerative medicine. Systems biology focuses on complex interactions within biological systems, using a holistic perspective, with the main aim of integrating all knowledge into a model and discovering emergent properties and networks to make it function as a system [1], [2].

Blastomeres from human pre-implantation embryos up to day-3 of development are considered to be totipotent since they can give rise to a complete embryo [3], [4]. From day 4 of development cells from the outside part of the embryo go on to form the trophectoderm, while the inside blastomeres generate the pluripotent inner cell mass (ICM) that will differentiate into mesoderm, ectoderm, and endoderm as well as the germ cells of the future human being [5]–[7].

Human embryonic stem cells (hESCs) are pluripotent cells that have been artificially created and do not exist in nature. They were initially derived from the ICM cells of the human blastocyst [8]–[11] but can also be obtained from other developmental stages, including single blastomeres from 5- to 8-cell embryos [12], [13]. hESCs represent an excellent model for regenerative medicine applications for the investigation of fundamental aspects of pluripotency. Indeed, the knowledge gathered from them was at the heart of the groundbreaking discovery of somatic cell reprogramming into a pluripotent state carried out by the overexpression of specific factors [14], [15].

For a short period of time the ICM is considered the paradigm of *in vivo* pluripotency. Indeed, for some time, cultured hESCs were considered to be equivalent to the ICM cells from which they were derived, although this concept was later revised [16]. In this context, recent studies have revealed that hESCs originate from a post-ICM intermediate, a transient epiblast-like structure which has undergone X-inactivation in female cells [17]. Furthermore, while blastomeres from day-3 embryos and the ICM share some biological similarities, they also exhibit significant differences as revealed by comparative gene expression analysis [18], [19].

Whole genome analyses are key to understanding the molecular mechanisms governing totipotency, and *in vivo* as well as *in vitro* pluripotency. Initial studies were performed by capturing a detailed view of hESC and ICM gene expression [16], [20]–[23], and further amplification protocols allowed single cell microarray analysis, thus making the profiling of gene expression in single blastomeres possible [16], [18], [24], [25]. Several differential gene expression studies have revealed that human blastomeres, ICM, and hESC signatures significantly differ [16], [18], [26], [27], suggesting the existence of independent developmental transcriptional signatures.

In this study, we aim to use these models from a systems biology perspective to investigate the inherent genomic signatures and networks governing human totipotency, and *in vivo* as well as *in vitro* pluripotency. Using this approach, we have also analyzed how pluripotent hESCs, regardless of their derivation source, might adapt to *in vitro* culture conditions.

## Results

### Comparative whole genome expression profile of human blastomeres versus ICMs and hESCs

**Figure 1:**
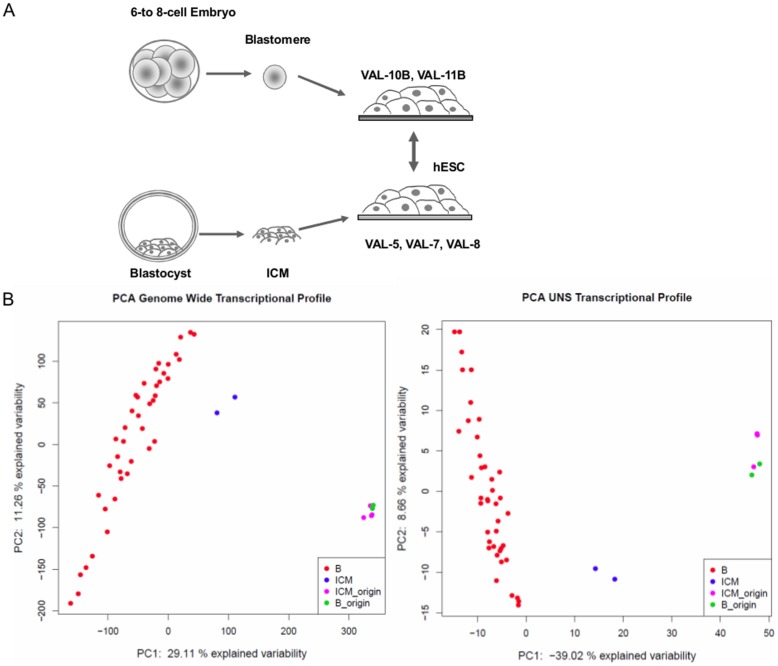
Experimental design of the study (A). Differential gene expression profile was carried out in single blastomeres from day-3 embryos, ICM from blastocysts, and their derived hESC counterparts. **Principal component analysis (PCA) of the whole transcriptome of single blastomeres from day-3 embryos, ICMs from blastocysts, and hESCs derived from both sources (B) and PCA of the UNS of blastomeres, ICMs, and hESCs (C).** Samples in the same cluster category stay closer together than in any other sample. The most separate clusters are blastomeres and hESCs, and ICMs show an intermediate pattern which falls between both. ICM_origin and B_origin correspond to hESC derived from ICM and Blastomeres respectively.

Human single blastomeres from day-3 embryos (6- to 8-cell stage; n = 41), ICM from human blastocysts (n = 2), three hESC lines derived from ICMs (VAL-5, -7, -8), and two hESC lines obtained from single blastomeres (VAL-10B, VAL-11B) were compared using genome-wide transcriptional analysis (Fig 1A). All hESC lines used in this study were derived in the same laboratory following the same protocol, and are fully characterized and registered (http://www.isciii.es/ISCIII/es/contenidos/fd-investigacion/fd-ejecucion/fd-programas-investigacion/fd-investigacion-terapia-celular-medicina-regenerativa/fd-banco-nacional-lineas-celulares/fd-lineas-celulares-disponibles/lineas-de-celulas-hES.shtml); VAL-5, -8 and -11B were XX, and VAL-7 and -10B were XY [11], [28], [29].

In order to compare the transcriptional profiles among them, microarray data were normalized and a principal component analysis (PCA) was carried out to identify clustering patterns that identified different origins (Fig 1B). Blastomeres and ICMs clustered into two separate groups while hESCs from different sources (blastomeres and ICMs) grouped to form a third cluster, indicating the existence of three transcriptional patterns, two according to developmental origin (blastomere and ICM), and a third according to the hESC phenotype, regardless their derivation origin. PCA revealed the existence of gene expression signatures that are indicative of single blastomeres (totipotency) ICMs (*in vivo* pluripotency), and hESCs (*in vitro* pluripotency). The relative position of these three PCA clusters informs of the similarities between their gene expression signatures, and indicates that blastomere and hESC differ the most while ICM occupies an intermediate position (Fig 1B). Furthermore, the differential gene expression profile showed no significant differences between individual hESC lines regardless their origin of derivation.

### Comparative Undifferentiation Network Signature (UNS) expression profile of human blastomeres versus ICMs and hESCs

To narrow down the genes and networks implicated in totipotency and pluripotency, we created an Undifferentiation Network Signature (UNS) composed of 266 genes characteristic of early human embryo developmental stages (blastomeres and ICM) and of hESCs. This selection was initially composed of 191 genes selected from published reports from our group and others that included the most characteristic undifferentiation markers, namely *NANOG*, *POU5F1*, *SOX2*, *GDF3*, *SOX2*, *DNMT3A*, etc. [16], [18], [21], [30]–[32]. Following this, an additional gene network analysis in the literature and databases (including GeneCards and iHOP) identified another 75 genes that highly interact with the genes included in our initial signature cohort, which were added with the purpose of understanding the gene pathways governing pluripotency (see Material and Methods). These connectors included transcription factors such as *WT1*, *TP53,* and *BMP* family members, adhesion molecules such as *CDH1*, or the transcription cofactor *EP300*. Housekeeping genes such as *GAPDH* and *ACTB*, and structural proteins like *NES* and *VIM* were also found in the connectors list, as well as several members of the MAP Kinase signaling pathway. Our final UNS included 266 genes composed of the most significant undifferentiation markers plus other markers which strongly interact with them, which overall, were involved in signaling pathways such as Activin/TGFB/BMP, Protein Kinases, FGF, Wnt, Retinoic Acid, Rho/Ras; transcriptional modulators related with telomerase activity; cell cycle, proliferation and self-renewal; cell adhesion, protein binding, and transport. Some of these genes are also related to epigenetic modifications, metabolism, cytoskeleton, or gamete differentiation. The detailed UNS gene list, including name, function, localization, global and specific pathways in which they are involved, as well as their relative expression in the models investigated is presented in Table S1.

Similar to the whole genome approach, we performed PCA to analyze interactions among the UNS transcriptional profiles which identified three distinct groups corresponding to single blastomeres, ICMs, and hESCs independently of their source of derivation (Fig 1C). As expected, the differences between these three groups was better captured by analyzing the UNS alone, as indicated by a larger variance on the first principal component (PC1) of the UNS PCA (39.02%) in comparison to the explained variance of PC1 in the global transcriptome analysis (29,11%) (Fig 1).

**Figure 2:**
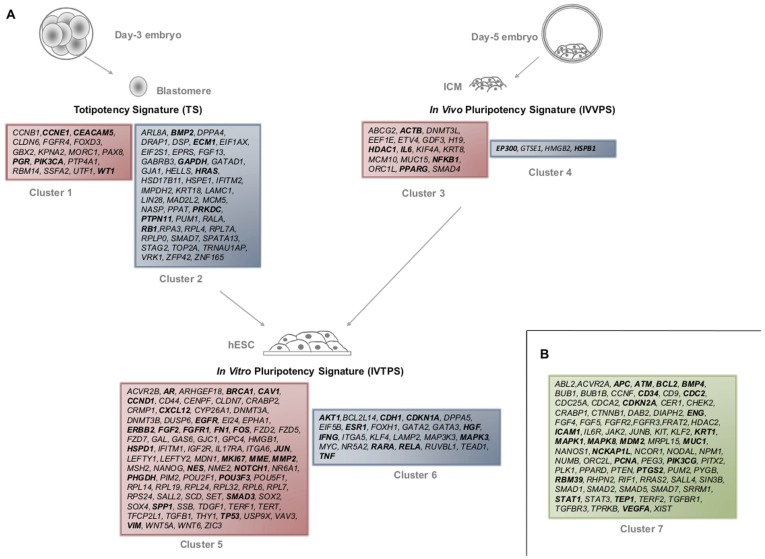
UNS genes grouping showing significant differences between single blastomeres, ICMs and hESCs. (**A**) Expression patterns with significant differences are grouped in six clusters according to over-expression or down-regulation after comparative gene expression analysis, giving rise to the totipotency signature (TS) from blastomeres (Clusters 1 and 2), the *in vivo* pluripotency signature (IVVPS) (Clusters 3 and 4), and the *in vitro* pluripotency signature (IVTPS). Red box means up-regulated genes, and blue box means down-regulated (Clusters 5 and 6). (**B**) Genes from the UNS with no significant differences in expression are also included (Cluster 7) and are represented in green. Connector genes are shown in bold. The criteria for statistically significant values was a p-value cutoff of <0.05. Complete data are described in Table S1.

Next, we grouped genes according to the differential expression level (up- or down- regulation) at each of the three cell types (single blastomeres, ICM and hESC). From the complete UNS, 189 genes showed significant differences between hESCs, ICMs and/or blastomeres (Fig 2A; clusters 1 to 6), while 77 genes did not (Fig 2B; cluster 7). Interestingly, the differential gene expression analysis revealed no significant differences between hESC lines originated from different sources of derivation, meaning that although single blastomere and ICM cells have very different gene expression profiles, their derived hESCs are transcriptionally equivalent, which points to a convergent adaptation to the *in vitro* culture conditions. Furthermore, the fact that 189 out of 268 UNS genes correspond to one cell-type defined cluster indicates that the great majority of the UNS genes are specific of a particular developmental stage (Fig 2A), highlighting the relevance of the cell-type specific gene signature.

### Totipotency Gene Signature (TS)

Differential gene expression of single blastomeres isolated from day-3 embryos versus ICMs and hESCs revealed a unique transcriptome signature that conforms to a totipotency signature (TS). The putative gene expression profile related to totipotency includes a group of 17 up-regulated genes and a group of 44 significantly down-regulated markers when compared to ICMs and hESCs. Up-regulated genes are mainly involved in cell adhesion (n = 2), cell cycle (n = 5), and regulation of transcription (n = 6) (e.g. *CCNB1*, *GBX2*, *WT1*, *FOXD3* and *UTF1*), whereas those down-regulated are involved in cell cycle (n = 8), signaling pathways (n = 11) and metabolism (n = 5) (e.g. *BMP2* or *GAPDH*), and mainly consisted of genes involved in cell proliferation (n = 10) and activation of transcription (n = 6) (e.g. *GABRB3*, *LIN28*, *ZFP42*, *TOP2A*, and *RPL*), as shown in Fig 2 and Table S2.

**Figure 3:**
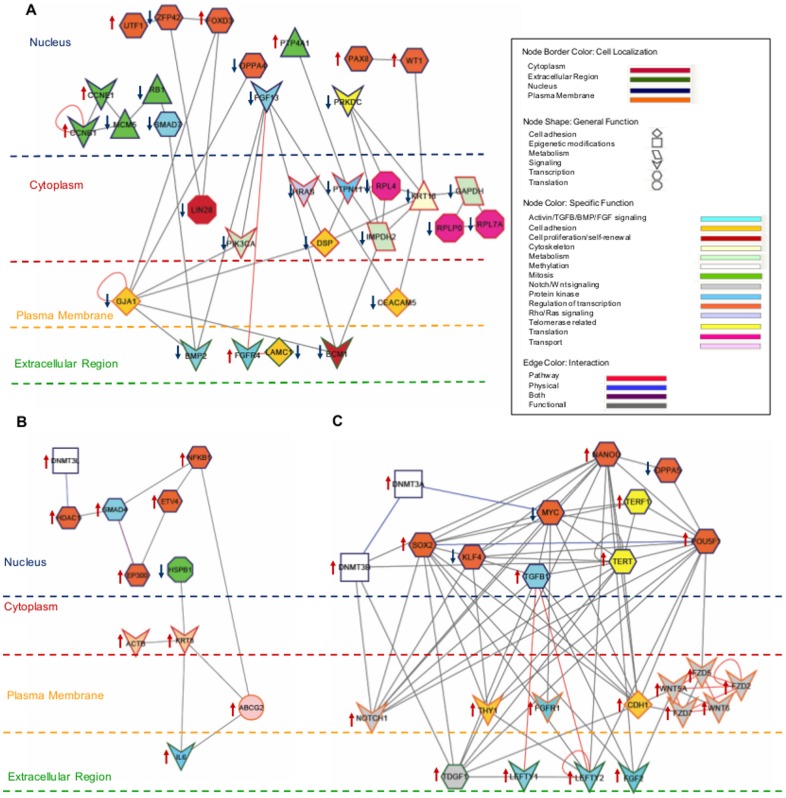
Cytoscape analysis of (A) TS, (B) IVVPS, and (C) IVTPS. Significant genes of each signature were represented according to their gene function and specific role in the cell, localization and type of interaction between them. Node border color refers to cell localization, node shape to general function and node color to specific function in the cell. Edge color refers to physical interactions, biochemical interactions or to both; when not specified, a functional interaction is assumed. Upstream arrow (red) means up-regulation versus the other categories, and downstream arrow (blue) means down-regulation versus the other categories. Microarray data values represented here are shown in Fig S1 and S2.

Differential gene expression results obtained for the TS were run using the web tool Cytoscape, an open source bioinformatics software platform for visualizing molecular interaction networks and biological pathways which integrates these networks with annotations, gene expression profiles and other data of interest [33]. Cytoscape analysis revealed 31 gene interactions of within the TS (Fig 3A) representing several networks. Interestingly, the transcription factors *UTF1*, *ZFP42*, and *FOXD3* directly connected in the nucleus, and the latter two also combined with the cytoplasmic proliferation molecule *LIN28*. Furthermore, *FOXD3* and *DPPA4* also directly interacted with the cell adhesion molecule *GJA1*. This protein located in the plasma membrane also showed direct interaction with signaling molecules in the cytoplasm and in the extracellular matrix, similar to the extracellular matrix protein (*ECMP*) or *BMP2* where other members of the FGF/activin and protein kinase pathways convey. Moreover, other genes proven to play key roles in undifferentiation such as WT1, have also been shown to directly interact with the cytoskeleton molecule *KRT18* which in turn directly binds to the adhesion molecule *CEACAM* and to molecules which play key roles in cell function like the housekeeping gene *GAPDH* and the ribosomal markers which form part of the TS (Fig 3A).

Moreover, all the components are tightly interconnected conforming to a global network where most components, from different locations in the cell, are eventually related. All TS components are extensively described in Table S2.

### *In Vivo* Pluripotency Signature (IVVPS)

The *In vivo* pluripotency signature (IVVPS) is composed of 21 genes that are significantly differentially regulated in ICMs versus single blastomeres and/or hESCs. Seventeen are over-expressed, including the protein membrane transporter *ABCG2*, the imprinting/methylation genes *H19* and *DNMT3L*, structural components like *ACTB* and *KRT8*, transcriptional factors like *GDF3* and *HMGB2*, cell adhesion molecules such as *MUC15*, signaling components like *IL6* and *SMAD4* and the receptor *PPARG* which is involved in important biological functions. Among the four genes down-regulated in ICMs versus blastomeres and hESCs are the cell cycle markers such as *GTSE1*, the transcription factor *HMGB2*, the heat shock protein *HSPB1*, and the transcriptional regulator *EP300* (Fig 2 and Table S3).

The Cytoscape analysis revealed that 11 markers interacted in the IVVPS (Fig 3B). All of them are up-regulated except the nuclear gene, *HSPB1* which is involved in mitosis. The methylation gene *DNMT3L* physically interacts with the transcription factor *HDAC1*, and directly binds to *SMAD4*. This protein kinase exhibits functional interaction with the transcription factor *NFKB1* and physical and biochemical interactions with the cofactor *EP300*. *NFKB1* directly interacts with the ABC transporter *ABCG2* which is also related to the extracellular interleukin, *IL6*, and they both join with the cytoplasmic cytoskeleton marker *KRT8*. The up-regulated gene *KRT8* functionally binds to the structural gene *ACTB*, and to the heat shock protein *HSPB1*, which is down-regulated in the *in vivo* pluripotency signature. All of these genes connect to finally form an intrinsic network describing the pluripotency signature *in vivo* (Fig 3B).

### *In Vitro* Pluripotency Signature (IVTPS)

*In vitro* pluripotency signature (IVTPS) is composed of 107 genes from the UNS which show significant differences between hESCs (derived from single blastomeres or isolated ICMs) versus these cellular sources prior to manipulation. This list of genes contains the most significant pluripotency and self-renewal markers, including the core pluripotency transcriptional genes, namely *POU5F1* (*OCT4*), *NANOG*, and *SOX2*. The list also contains some other important transcription factors (*HMGB1*, *KLF4*, *MYC*, *SALL2* etc.), the telomerase related genes, *TERT* and *TERF1*, ‘*de novo*’ methylation markers (*DNMT3A*, *DNMT3B*), genes belonging to the most representative pluripotency signaling pathways such as Activin/TGFB/BMP (*BMP2*, *LEFTY1*, *LEFTY2*, *TGFB1*), Wnts (*WNT5A*, *WNT6*, *TDGFB1*), and ribosomal genes (*RPL14*, *RPL7*, *RPS24*), among others (Fig 2). The complete list of the 107 genes constituting the hESC signature is detailed in Table S4 along with their main characteristics and properties. Although the majority were represented by previously reported genes, more than 30% of them are new interacting molecules, including the cytoskeleton proteins *VIM* and *NES*, the transcription factors *TP53* and *FOS*, and cell proliferation proteins *AR* and *CXCL12* (Table S4). All these genes show a high degree of interaction, as represented by Cytoscape analysis, where only *PIM2* remains unbound (Fig S2). Differential gene expression analysis revealed that almost 80% of the genes that conform to the IVTPS are over-expressed in hESCs when compared to blastomeres and ICM, suggesting transcriptional machinery activation.

Network analysis was also run for the most representative undifferentiating markers, highlighting the IVTPS and the presence of interacting markers that form a straight network, which may help us to understand and complete their signaling pathways (Fig 3C), and confirming previous studies in which direct interactions between transcription factors as *POU5F1*, *NANOG*, *SOX2* and *MYC* are reported [31], [34], [35]. Cytoscape analysis shows how an adhesion interacting molecule located on the plasma membrane, *CDH1*, functionally interacts with the most important transcription factors that characterize pluripotency and self-renewal in the nucleus, namely *POU5F1*, *NANOG*, *TERT*, *TGFB1*, *SOX2* (up-regulated in hESCs versus single blastomeres and isolated ICMs), and *MYC* and *KLF4* (down-regulated in hESCs versus single blastomeres and ICMs), with two components of the Wnt signaling network (*WNT5A* and *FZD7*) in the nucleus, and with *TDGF1*, from the same signaling network but in the extracellular space. Thus, these interactions describe new functional interactions and putative regulatory networks controlling hESCs *in vitro*. *DPPA5*, is up-regulated in single blastomeres and ICM cells and directly binds with *POU5F1* and *NANOG*, suggesting that direct regulation may occur. The ‘*novo* methylation’ gene *DNMT3A* physically joins to the down-regulated transcription factor *MYC* and to *DNMT3B* in the nucleus. Interestingly *DNMT3B* functionally interacts with the up-regulated transcription factor *SOX2*, the telomerase related gene *TERT1* in the nucleus, with *NOTCH1* in the plasma membrane, and with *TDGF1* in the extracellular space (Fig 3C). These results show a complex and intricate network, in which multiple components with different functions and from several different signaling pathways are connected in different parts of the cell.

### Signatures Comparison and Functional Enrichment Analysis

Gene signatures from each category, TS, IVVPS, and IVTPS obtained from the UNS gene expression clustering analysis were further analyzed. The distribution of the connector genes in each signature was also studied. Interestingly, almost 70% of interacting genes were shown to be present in any of the three signatures, suggesting a putative active role in controlling each signaling process, and confirming that these new molecules are facilitators present in all the signatures we have identified (Fig 2).

**Figure 4:**
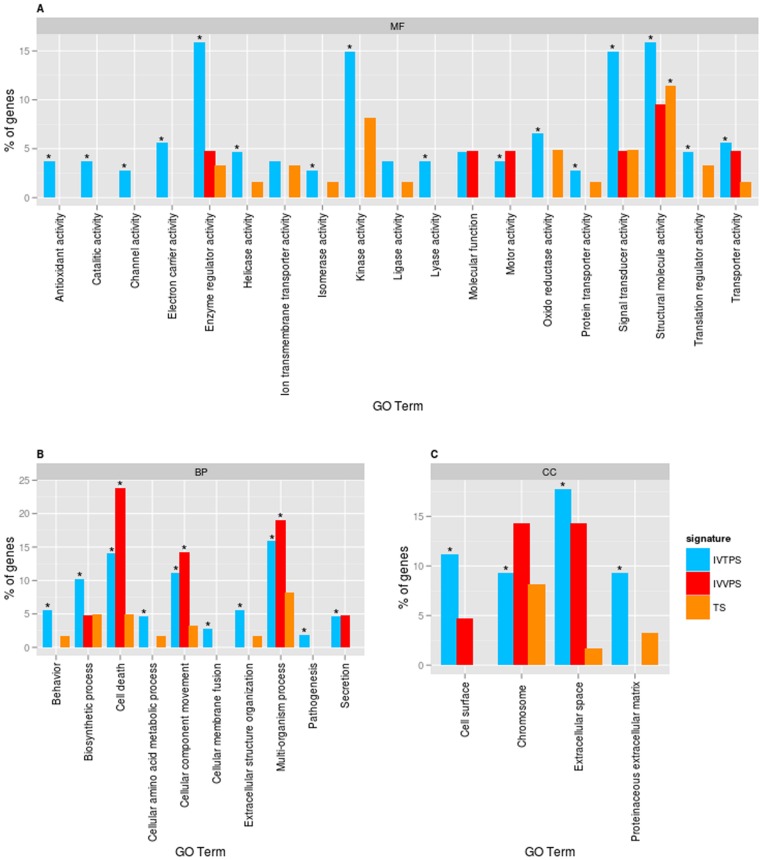
Gene Ontology of functional comparison between TS, IVVPS, and IVTPS. GO slim GOA analysis of the three ontologies are represented separately: (**A**) Molecular Functions (MF); (**B**) Biological Process (BP); and (**C**) Cellular Components (CC). Each GO term from a GOslim subset is represented on the x-axis, and gene content in percentage related to each gene signature is compared on the y-axis. The asterisk marks the over-represented significant terms (GOSlim GOA adjusted-p-value <0.05) in gene signatures after Fisher exact test genome comparison.

Functional enrichment allows a statistical approach for genes belonging to a Gene Ontology (GO) category. The functional enrichment of UNS was achieved by comparing gene signatures between the UNS versus the complete genome, each signature versus the UNS, each signature versus the complete genome, and between them using the functional enrichment tool FatiGo, from the Babelomics platform. The functional enrichment analysis of the UNS versus complete genome comparison resulted in a very large number of enriched terms, even applying a very restrictive p value (p value<0.005); 1,630 Biological Process (BP), 452 Molecular Function (MF), and 155 Cell Component (CC) GO terms, as well as 121 KEGG pathways. To visualize and summarize functional results among signatures GOslim GOA gene distribution terms were obtained, representing each signature compared to the whole genome (Fig 4).

The IVTPS showed the highest number of significant terms in the whole GOslim GOA search term analysis, MF like those mentioned as antioxidant, catalytic, channel or electron carrier activities were present only in this signature, while some other MF such as kinases, signal transducers and molecules with structural activities were also found to be enriched (Fig 4A). Some enriched BP terms included cellular membrane fusion, and extracellular structure organization, reaction to external or internal stimuli (behavior) and biosynthetic processes, among others (Fig 4B). Furthermore, CC cohort analysis also showed that terms that were mainly related with cell surface, extracellular space and proteinatious extracellular matrix components (Fig 4C) were enriched. GO terms analysis was in concordance with IVTPS molecules and their expression pattern, in which many of up-regulated genes were described as playing key roles not only in transcription but also in signaling and secretion (Fig 2 and 3), and might also play a role for *in vitro* culture survival. When GOslim was applied for down-regulated genes in the IVTPS, all 33 terms were found significant for IVTPS as GATA2 and GATA3 are present in all terms, thus non supporting differential information. The IVVPS was also enriched in enzyme and structural molecular activity regulating genes as well as those involved in transporter activity (Fig 4A). In terms of the BP cohort, IVVPS genes were enhanced in processes related to cellular component movement, secretion, multi-organism processes and cell death (Fig 4B). CC analysis showed a protein distribution occupying chromosomes, cell surface and extracellular space (Fig 4C), which correlates with the genes included in the IVVPS signature which included mainly up-regulated transcription factors, cytoskeleton components, and a gene transporter (Fig 2 and 3). No significant terms were been found for down-regulated genes in the IVVPS in the GOslim GOA analysis. Finally, TS components were mainly enhanced in activities related with structural molecules, oxidoreductases and kinases in the MF analysis (Fig 4A); no specific BP molecules were over-represented compared to IVVPS and IVTPS, although multi-organism process, cell component movement and behavior BP components were enhanced (Fig 4B). CC analysis of the TS showed a general distribution throughout all the categories excluding the cell surface (Fig 4C). When down-regulated genes in TS signature were functionally analyzed, two terms, structural molecule activity and chromosome were found to be enriched.

Every gene list comparison was also analyzed for pluripotency versus the UNS, totipotency versus the UNS and pluripotency versus totipotency. No significant results were found (adjusted p-value <0.1) in any of these comparisons, with the exception of the TS versus IVTPS, where the cell surface term (GO: 0009986) was over-represented (adjusted-p value = 0.054629), and included the 16 following genes: *ACVR2B*, *BRCA1*, *CAV1*, *CD44*, *GATA2*, *GATA3*, *HSPD1*, *ITGA5*, *ITGA6*, *KLF4*, *RARA*, *RELA*, *TDGF1*, *TGFB1*, *THY1*, *TNF*. These results concur with previous results, strongly supporting the concept of hESC culture adaptation.

## Discussion

hESCs can be derived from different sources, which include single blastomeres from day-3 embryos, and the ICM from blastocysts. These different developmental origins led us to hypothesize that different using starting material from different sources could result in hESC that have distinctive gene expression profiles. With that purpose, we took a genome wide approach to compare the genome expression profiles of human blastomeres versus ICMs and hESCs. PCA identified three transcriptional patterns, two according to developmental origin (blastomere or ICM), and the third corresponding to hESCs, regardless their derivation origin.

Thus, the concept of totipotency (TS), *in vivo* and *in vitro* pluripotency (IVVPS and IVTPS) was further investigated using a systems biology approach, and with the help of an undifferentiation network signature (UNS). The UNS was created to elucidate global signaling pathways that control each developmental stage and was formed by compiling genes that had previously been reported to play key roles in undifferentiation (n = 191) and combining them with genes that strongly interact with them, termed as “connectors” (n = 75). Comparative gene expression analysis was applied to the UNS and confirmed our previous PCA results, allowing us to establish a gene clustering unique to each developmental signature. When functional enrichment was applied to our UNS, a large number of general GO functions were identified, which is expected for such a signature created from bibliographic and database resources. Connector molecules are mostly signaling and housekeeping genes that play a fundamental role in basal cellular functions. These connectors represent the 20%, 33%, and 31% of the TS, IVPS and IVTPS respectively, also indicating the relevance in defining the function of the each stage specific functions.

The TS was created from samples originating from single blastomeres from day-3 embryos, which resulted in a specific signature constituted by 61 genes; 28% of which were up-regulated and principally comprised cell cycle markers, signaling components, and transcription factors, such as *GBX2* and *UTF1* both implicated in maintaining an undifferentiated state cost commonly by gene repression [36], [37]; 72% were down-regulated, including the transcription factors *WT1* and *FOXD3* [38], [39]. Down-regulated markers also included transcription factors involved in cell transcription, proliferation, pluripotency and telomerase activity such as *GABRB3*, *IFITM2*, *ZFP42* and *PRKDC* [21], [40], [41], *RPL4*, *RPL7A*, *LIN28* [42], and genes involved in cell metabolism such as *GAPDH* and *IMPDH2* [43], [44]. Furthermore, network analysis revealed a highly interconnected association with more than 50% of the TS genes showing at least one interaction, and in which transcription factors, signaling, translational, and structural components, as well as molecules involved in metabolism and cell adhesion were interconnected. This low expression profile may be indicative of a transient developmental stage in which single blastomeres may be preparing for later developmental changes, and is in concordance with GO analysis in which several MF and BP terms are enriched, albeit in a lower proportion than in the other gene signatures discussed.

The ICM gene expression profile is represented by the IVVPS, and is constituted by very few genes (n = 21), 80% of them were over-represented and 20% were down-regulated. Most of the up-regulated markers play key roles in the cell, such as *MCM10* which is involved in the initiation of genome replication [45] and *DNMT3L* which stimulates *de novo* methylation and also mediates transcriptional and epigenetic repression through its interaction with *HDAC1* [46], [47], and the cytoskeleton components *ACTB* and *KRT8*. The ABC transporter, *ABCG2*, is also included in this group, and is notable for its selectivity in separating putative stem cells by cell sorter assays [48], [49]. Down-regulated genes included the heat shock protein *HSPB1* which inhibits translation [50], and the recently described pluripotency cofactor, *EP300* [51]. Most of the markers mentioned above show physical, biochemical and functional interactions that may control the *in vivo* pluripotency of ICM cells. Furthermore, functional analysis showed that the CC components included chromosome, cell surface and extracellular space terms enriched in the IVVPS, supporting the notion of an enhanced role for cell membrane transport and trafficking in ICM cells. This has also been confirmed by similar enrichment in the BP category cellular components also involved in movement, multi-organism process, secretion and cell death, and the MF enzyme regulatory, motor, signal transducer and transporter activity GO terms.

Finally, the IVTPS is unique to hESC, and is composed of the most numerous group of genes in our UNS (n = 107). Of these, 78% were up-regulated and 22% down-regulated. Up-regulated genes in hESCs versus ICMs and blastomeres included the most significant markers of pluripotency and cellular immortality characterisation, namely the transcriptional core *NANOG*, *POU5F1* (*OCT4*), and *SOX2*, and telomerase related *TERT1* and *TERF1*, as well as Activin/Nodal signaling markers such as *TGFB1*, *LEFTY1* and *LEFTY2*, Wnt signaling such as *WNT5A* and *WNT6*, adhesion molecules such as *THY1*, ribosomal genes involved in cell proliferation (*RPL6*, *RPL14*), and the transcriptional repressor *TP53* [21], [30], [31], [52], [53]. The DNA methyltransferases, *DNMT3A* and *DNMT3B*, which play key roles in regulating gene expression and chromatin structure [54], [55] were up-regulated in hESCs, as shown in previous studies, and confirmed by these results [22], [56], [57], while the enzyme catalysing their activity, *DNMT3L*, was up-regulated in the IVVPS. *KLF4* and *MYC* genes, which have been used for reprogramming somatic cells in order to obtain induced pluripotent stem cells (iPSCs) [14], [15], have been found to be down-regulated in hESCs when compared to single blastomeres and ICMs, suggesting that these genes may be necessary in exceedingly high levels for the induction of pluripotency, and at lower levels during propagation. Indeed, it has been reported that *MYC* participates in facilitating undifferentiation by stimulating gene expression and proliferation [51], [58].

Network analysis revealed a high degree of interconnection between all IVTPS cohort members. Indeed, only one of the 107 genes in the signature showed no interaction with any other gene. Most members showed 4 or 5 interactions with other transcription factors, molecules involved in signaling, adhesion, metabolism or translation (Fig S2). In fact, previous studies have reported that the core transcription factors *OCT4*, *SOX2*, and *NANOG* may control reprogramming both by positively regulating their own promoters, forming an interconnected self-regulatory loop, and secondly by co-occupying and activating the expression of genes necessary for maintaining an undifferentiated stage, while contributing to the repression of genes that code for differentiation routes [31], [35], [53]. In general terms, it has been described that biological networks consist of highly connected nodes called hubs, which if removed would lead to fragmentation of the network [51], [59]. Some of the genes that constitute hubs receive extensive inputs. For example, the enhancer region of the *Oct4* gene is bound by at least 14 transcription factors in mouse ESCs (mESCs) [59], and our networking analysis reveals that human *OCT4* (*POU5F1*) shows direct interactions with 29 markers of the *in vitro* pluripotency signature, 16 from the nucleus (*GATA3*, *KLF4*, *MIKI67*, *SOX2*, *ESR1*, *MYC*, *CCND1*, *HMGB1*, *RELA*, *POU2F1*, *TERT*, *TERF1*, *DPPA5*, *NR6A1*, *RUVBL1*, *TP53*), 3 from cytoplasm (*NES*, *VIM*, *GAS6*), 5 from the plasma membrane (*FZD5*, *NOTCH1*, *THY1*, *CDH1*, *CD44*), and 5 from the extracellular space (*LEFTY2*, *TDGF1*, *FN1*, *EGFR*, *TNF*) (Fig S2). These interactions serve as key regulators in the enhancement of transcription, as genes bound by more transcription factors tend to be more actively transcribed [59]. This model could be additionally applied to human gene regulation, in which the more transcription factors forming the hub are occupied, the higher the gene expression, especially when combined with the idea that the key pluripotency-associated factors may self-regulate their own expression [59]. In the same context, *TERT1* and *TERF1*, which mediate self-renewal and cellular immortality, have also been shown to be up-regulated in hESCs, as previously demonstrated with *TERF1* in mice [60].

All these results suggest that hESCs might adapt to cell culture conditions, by activating a vast number of transcription factors, signaling, cell adhesion, cell proliferation, and translation molecules. This putative *in vitro* adaptation is supported by functional analysis which revealed a high number of MF category GO search terms in the IVTPS cohort, corresponding to cellular transport, signaling and enzymatic activities, and general molecular and structural function. Enriched BP category terms included those related to stimuli response, cellular component movement and secretion, as well as metabolic processes, extracellular structure organization and cellular membrane fusion. Enhanced CC group terms were generally distributed evenly over GO term cohorts, although cell surface and extracellular matrix and space terms were enhanced. All these enriched terms encompass genes that are mainly related to the cell adhesion and interaction necessary for hESCs to form their characteristic colony structure in culture for adapting to *in vitro* cell culture conditions. Therefore, hESCs display a common gene profile, independent of the source of derivation, which is different from that of single blastomeres, and ICM cells. These results concur with recent reports showing that hESCs derived from different stages of embryo development exhibit very little difference in their gene expression profiles, maintain a similar pluripotent phenotype, and that the slight differences observed are probably due to differences in derivation and culture procedures [5]. In our case, variability due to experimental procedures was minimized, thus leading us to conclude that there were no significant differences between hESCs from different developmental sources. These results could be of great importance to the understanding of human embryo development, pluripotency, and reprogramming of somatic cells to iPSCs.

Overall, in this paper we present a defined gene signature for blastomeres (TS), hESCs (IVTPS), and ICMs (IVVPS). Network analysis allowed us to establish biochemical, physical, and functional interactions between the genes that segregated to each expression cluster, which may define each developmental regulation stage. This was enabled by a systems biology approach, which allows the integration of massive data from biological databases with classical molecular biology information in a cellular and physiological context that accurately approximates the real situation occurring in biological processes [61]. This new perspective is resulting in a revolution in classical molecular biology approaches and will enable us to continue to elucidate complex processes and pathways, such as those related with the beginning of human embryo development.

## Materials and Methods

### Experimental Design

Transcriptional profiles from Day-3 embryos single blastomeres, and from ICM cells [18] were compared to the hESC signature obtained from single blastomeres from Day-3 embryos, and to ICM cells from blastocysts, namely: VAL-10b and VAL-11b; and VAL-5, -7, and -8, respectively [11], [28], [29] (Fig 1A).

### Ethical Permission

Permission for this Project was granted by the Spanish Authority, *Instituto de Salud Carlos III* on December 13^th^ 2006 for the project entitled “Derivation of human embryonic stem cells (hESC) of therapeutic grade in Spain.” Human embryos frozen at different stages at the *Instituto Valenciano de Infertilidad* (*IVI*) were donated for this work according to the Spanish law 45/2003. The donors were asked to sign a specific consent form for stem cell derivation as indicated in the *Real Decreto* 2132/2004. All hESCs used in this work have been characterised, published and registered in the Spanish Stem Cell Bank (www. isciii/htdocs/terapia/terapia_bancocelular.jsp) and are available worldwide. This work was performed in the Valencian Node of the Spanish Stem Cell Bank, at the Centro Investigación Principe Felipe (CIPF).

### Derivation of hESCs

#### From whole embryos

Donated frozen embryos were thawed using an Embryo Thaw Kit (Vitrolife, Kungsbacka, Sweden) according to the manufacturer's instructions. Pronuclear stage and day-two embryos was transferred to IVF and CCM medium 1∶1 (Vitrolife). Thawed day-3 embryos were transferred to CCM medium (Vitrolife) and cultured for an additional 2 to 3 days. At the blastocyst stage, the zona pellucida was removed by treatment with Tyrode's acid solution, after which the embryos were sequentially washed in CCM and HES medium.

Zona-free blastocysts were cultured on irradiated human foreskin fibroblasts (ATTC) in multiwell cell culture plates (Beckton, Dickinson and Company, Erembodegem, Belgium) in 80% Knockout DMEM (Gibco/BRL, Paisley, Scotland, UK), 20% Knockout SR (Gibco/BRL), 1 mM glutamine (Sigma, St. Louis, MO), 0.1 mM β-Mercaptoethanol (Sigma), 1% non-essential amino acids stock (Gibco/BRL), 20 ng/ml of human basic Fibroblast Growth Factor (h-bFGF) (Invitrogen, Life Technologies, Carlsbad, CA) and 0.5% Penicillin-Streptomycin (Sigma). The plate was incubated at 37°C and 5% CO_2_ with no manipulation for 3 days. After this period, the medium was changed every 48 h and the culture was maintained for 2–3 weeks, until outgrowth with hESC morphology appeared. The outgrowth was dissociated mechanically avoiding the areas corresponding to trophoectoderm. The isolated fragments were re-plated in a new well containing new irradiated feeder cells and fresh HES medium. The medium was changed every 48 h and the growth of colonies with hESC morphology was checked under microscope. When several colonies were expanded the cryopreservation and characterisation of the new cell line was performed. This process was followed for VAL-5, -8, and -9 [11], [28].

#### From Inner Cell Masses isolated with a laser

Once the embryo was thawed and cultured until blastocyst stage, the inner cell mass (ICM) was isolated using a micromanipulator. The holding micropipettes (Humagen, Charlottesville, VA) were put in the micromanipulator on the inverted Microscope. The blastocyst was placed in a drop of GPGD medium (Vitrolife) supplemented with 5% Human Serum Albumin (Vitrolife) in a micromanipulation plate (Becton, Dickinson, and Company) and was held with holding pipettes from both sides, trying to localise the ICM at 9 o'clock position. The ICM was separated from trophectoderm by laser shots cutting perpendicularly to the pipettes, from up to down as near as possible to ICM whilst avoiding its damage. When both parts were separated, the zona pellucida was separated by careful pipetting. The isolated ICM was seeded on irradiated human foreskin fibroblasts (ATTC) and the protocol described above was followed. This protocol was followed for VAL-7 [28].

#### From single blastomeres

Donated frozen day-3 human embryos were thawed and incubated in CCM medium (Vitrolife) for at least 3 h under standard culture conditions (37°C, 5% CO_2_). Then, the single blastomere was removed from the embryo by a biopsy procedure similar to that it used in pre-implantation genetic diagnosis of genetic defects. The biopsied embryo was transferred to CCM medium (Vitrolife), cultured for additional 2–3 days and at blastocyst stage was cryopreserved.

The biopsied blastomere was transferred into a drop of CCM medium covered with mineral oil (Sigma) and cultured for 24 h. The isolated blastomere was transferred onto irradiated human foreskin fibroblasts (ATTC) in drops of CCM medium (Vitrolife) supplemented with 10 µg/ml human Laminin (Sigma) and covered with mineral oil (Sigma), this was referred to as day 0. From day 3, the medium drop containing the attached blastomere was refreshed daily by replacing ^1^/_3_ of the volume with CCM medium supplemented with human Laminin (Sigma; 10 µg/ml) and 25 ng/ml of h-bFGF (Invitrogen, Life Technologies, Carlsbad, CA). From day 5, CCM medium was replaced with standard hESC medium (80% KnockOut-DMEM, 20% KnockOut Serum Replacement, 25 ng/ml bFGF) enriched with 10% FCS, and replaced in drops on a daily basis. When an initial hESC colony was detected, it was dissected one or two days later within the same drop. The procedure was repeated in approximately five days. After the second dissection, small hESC clumps were transferred into a 4-well dish (Nunc) with freshly seeded irradiated human feeders. The following day, the FCS was withdrawn from the medium and replaced with the standard serum-free hESC medium. This process was followed for the derivation of VAL-10B and VAL-11B [29].

### Culture and maintenance of undifferentiated hESCs

All hESC lines were cultured and maintained on irradiated human foreskin fibroblasts (ATTC) in multiwell cell culture plates (Becton, Dickinson, and Company, Erembodegem, Belgium) in 80% Knockout DMEM (Gibco/BRL, Paisley, Scotland, UK), 20% Knockout SR (Gibco/BRL), 1 mM glutamine (Sigma, St. Louis, MO), 0.1 mM β-Mercaptoethanol (Sigma), 1% non-essential amino acids stock (Gibco/BRL), containing 10 ng/ml of bFGF (Invitrogen, Life Technologies, Carlsbad, CA). The colonies were incubated at 37°C with 5% CO_2_, and the medium was changed every 48 h.

The colonies were mechanically dissected into clumps every 4 to 5 days and transferred to dishes containing new inactivated human foreskin feeder cells.

### Preparation of RNA for microarray analysis

The RNA from hESCs was isolated using Zymo Research's Mini RNA Isolation Kit™ (Zymo Research Corporation) following the manufactureŕs recommendations. A total of 100,000 cells per VAL line were used for the RNA extraction destined for microarray analyses.

### Microarrays

RNA was quantified by spectrometry (NanoDrop ND1000, NanoDrop Technologies, Wilmington, Delaware USA) and the quality confirmed using an RNA 6000 Nano Kit and Bioanalyzer (Agilent Technologies, Palo Alto, California USA) assay. 480 ng of total RNA was used to produce Cyanine 3-CTP-labeled cRNA using the One-Color Quick Amp Labelling Kit (Agilent p/n 5190-0442) according to the manufacturer's instructions. Following ‘One-Color Microarray-Based Gene Expression Analysis’ protocol Version 5.7 (Agilent p/n G4140-90040), 3 µg of labelled cRNA was hybridised with the Whole Human Genome Oligo Microarray Kit (Agilent p/n G2519F-014850) containing 41,000+ unique human genes and transcripts. Arrays were scanned in an Agilent Microarray Scanner (Agilent G2565BA) and data were extracted using Agilent Feature Extraction Software 9.5.3 following the Agilent protocol GE1-v5_95_Feb07 and the QC Metric Set GE1_QCMT_Jan08.

Microarrays from single biopsied blastomeres, ICMs, and hESCs from both sources are deposited under GEO numbers GSE22032 and GSE42520.

### Bioinformatic analysis

Gene expression data was analysed using the Limma Bioconductor package [62]. The Normexp function was applied for background correction, quantile normalization [63] was used to standardize across arrays and multiple testing adjustment of p-values was done according to Benjamini and Hochberg's methodology [64].

For gene network analysis, an undifferentiation network signature (UNS) was created using genes previously reported in the literature to be characteristic of undifferentiation (n = 191) [16], [18], [21], [30]–[32] which were combined with genes that strongly interact with them, referred to as connectors (n = 75). These interaction data were obtained from the iHOP database [65] and GeneCards V3 database [66]. Connector genes were included in the UNS only if they had at least 15 interaction partners within the initial 191 undifferentiation gene list. Data processing was done using in house R scripts. The UNS was further visualized and analyzed in Cytoscape [33] where genes were labeled according to cell localization, molecular function, and the signaling or metabolomic pathways they are involved in. This functional data was obtained from BioMart database.

Functional enrichment was performed with FatiGO [67] a widely used SEA implementation, which is included in the Babelomics [68] web-based package using the Gene Ontology (GO) [69], and KEGG Pathways [70] vocabularies. GO term annotations for the genes in the microarray were taken from the Ensembl database (release 55), and KEGG Pathway annotations were obtained from the KEGG web page. GO slims are cut-down versions of the GO ontologies which contain a subset of the terms in the whole GO ontology library. They give a broad overview of the ontology content without the specific fine grain details of the terms which is particularly useful for giving a summary of the GO annotation results. The GOslim options were selected in FatiGO in order to summarize functional enrichment comparison among signatures. For functional enrichment analysis, GO slim GOA, from FatiGO enrichment tool from Babelomics, was eventually used. GOslim GOA is composed by 33 terms, and all are used, hence no level needs to be selected.

### Validation by real-time quantitative PCR (qPCR)

cDNA retro-transcription was performed with the MMLV enzyme contained in the Advantage™ RT-for-PCR kit (Clontech, Takara, Japan) using oligo (dT)_18_ as primers. 0.5–1 µg of total RNA was subjected to initial denaturalisation, retro-transcription for 60 min at 42°C and final enzyme inactivation at 70°C for 10 min.

For qPCR experiments, 150 ng of synthesised cDNA of each VAL line studied was included [71]. Experiments were performed in duplicate, and each lot of experiments included an internal positive control and a negative water control. 2 µl of cDNA was added to each qPCR reaction which was carried out using the LightCycler FastStart PLUS Master SYBR Green (Roche) in a LightCycler 2.0 (Roche). qPCR cycles consisted of one denaturalization step at 95°C for 10 min, one amplification step of 40 cycles of 95°C 10 secs, 59°C, 6 secs, and 72°C 10 sec, and a melting curve to assess amplicon specificity. Validation assays were performed for DPPA5, *HMGB1*, *MYC*, *POU5F1*, *RPL14*, and *RPL19* genes in non-amplified samples. Results are shown in Fig S3.

## Supporting Information

Figure S1 **Cytoscape analysis showing microarray data value representation of: (A) the totipotency signature (TS), (B) the *in vivo* pluripotency signature (IVVPS), and (C) the selected *in vitro* pluripotency signature (IVTPS).**
(TIFF)

Figure S2 **(A) Cytoscape analysis of all gene markers showing any interaction from the** ***in vitro*** **pluripotency signature.** Node border color refers to cell localization, node shape to general function, and node color to specific function in the cell. Edge color refers to physical interactions, biochemical interactions or to both; when not specified functional interaction is assumed. Upstream arrow (red) means up-regulation versus single blastomeres and ICM, and downstream arrow (blue) means down-regulation versus blastomeres and ICMs. **(B) Microarray data value representations of the** ***in vitro*** **pluripotency signature markers showing any interactions.**
(TIFF)

Figure S3 **Validation of microarray results by real-time quantitative PCR.** (A) Results obtained from qPCR analysis performed on non-amplified blastomeres, ICMs, and hESCs (VAL-5,-7, -8, 10B, -11B) for *DDPA5*, *HMGB1*, *MYC*, *POU5F1*, *RPL14* and *RPL19*. *RPS24* were used as references. (B) Microarray data corresponding to genes analyzed.
(TIFF)

Table S1 **Gene list of the Undifferention Network Signature (UNS).** Genes were added for their high interaction ratios and the databases used are indicated. Gene name, description, cell localization, and main and specific function in the cell are also specified. Normalized data values for each category, single blastomeres from day-3 human embryos, ICMs from blastocysts, hESCs derived from single blastomeres, and hESCs derived from ICMs. Normalized data are log-transformed expression values. The criteria for statistically significant values was a p-value cutoff of <0.05
(XLS)

Table S2 **Gene list constituting the totipotency signature (TS).** Gene name, description, cell localization, and main and specific function in the cell are also specified. Normalized data values for each category, single blastomeres from day-3 embryos, ICMs from blastocysts, hESCs derived from single blastomeres, and hESCs derived from ICMs. Normalized data are log-transformed expression values. The criteria for statistically significant values was a p-value cutoff of <0.05.
(XLS)

Table S3 **Gene list constituting the** ***in vivo*** **pluripotency signature (IVVPS).** Gene name, description, cell localization, and main and specific function in the cell are also specified. Normalized data values for each category, single blastomeres from day-3 embryos, ICMs from blastocysts, hESCs derived from single blastomeres, and hESCs derived from ICMs. Normalized data are log-transformed expression values. The criteria for statistically significant values was a p-value cutoff of <0.05.
(XLS)

Table S4 **Gene list constituting the** ***in vitro*** **pluripotency signature (IVTPS).** Gene name, description, cell localization, and main and specific function in the cell are also specified. Normalized data values for each category, single blastomeres from 6- and 8-cell embryos, ICMs from blastocysts, hESCs derived from single blastomeres, and hESCs derived from ICMs. Normalized data are log-transformed expression values. The criteria for statistically significant values was a p-value cutoff of <0.05.
(XLS)
