# Supplementary figures and images for: Defining the Genomic Signature of Totipotency and Pluripotency during Early Human Development
Source: PLoS One. 2013 Apr 17;8(4):e62135. doi: 10.1371/journal.pone.0062135 (PMC3629124; doi:10.1371/journal.pone.0062135)

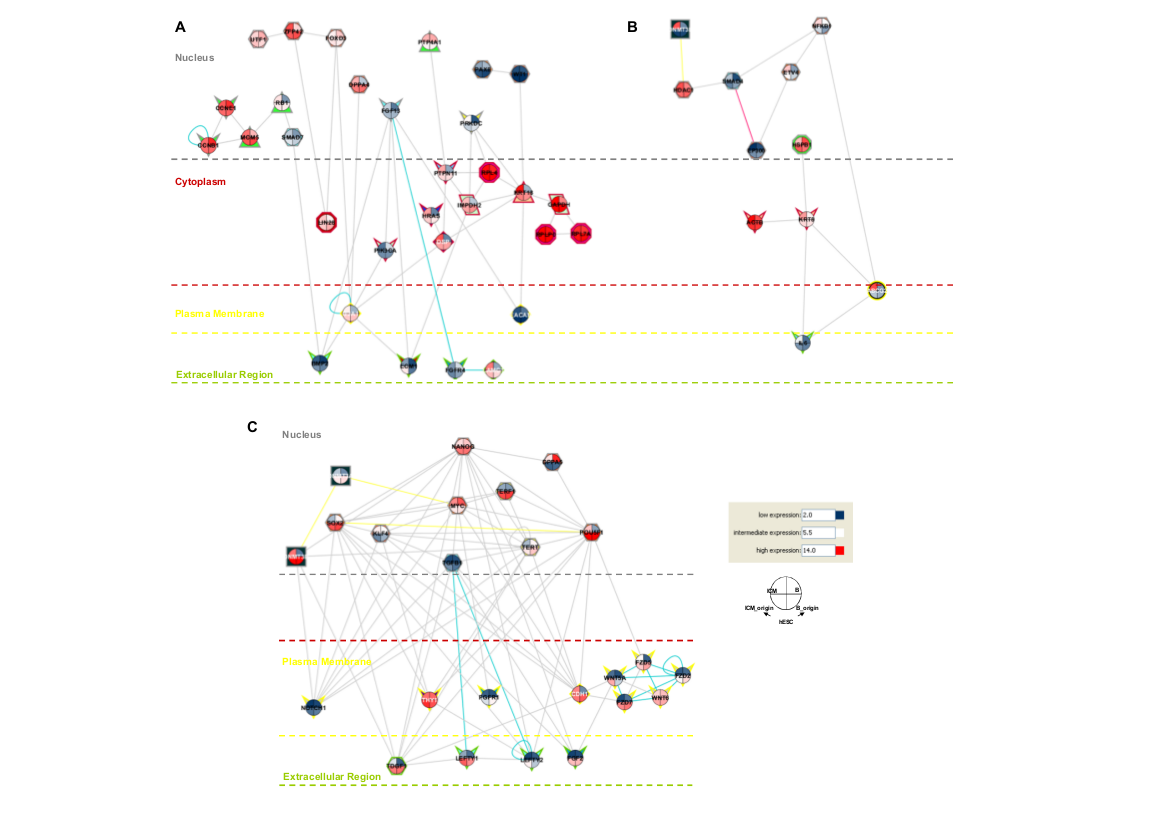

Supplement: Figure S1 — Cytoscape analysis showing microarray data value representation of: (A) the totipotency signature (TS), (B) the in vivo pluripotency signature (IVVPS), and (C) the selected in vitro pluripotency signature (IVTPS). (TIFF) [file pone.0062135.s001.tiff]

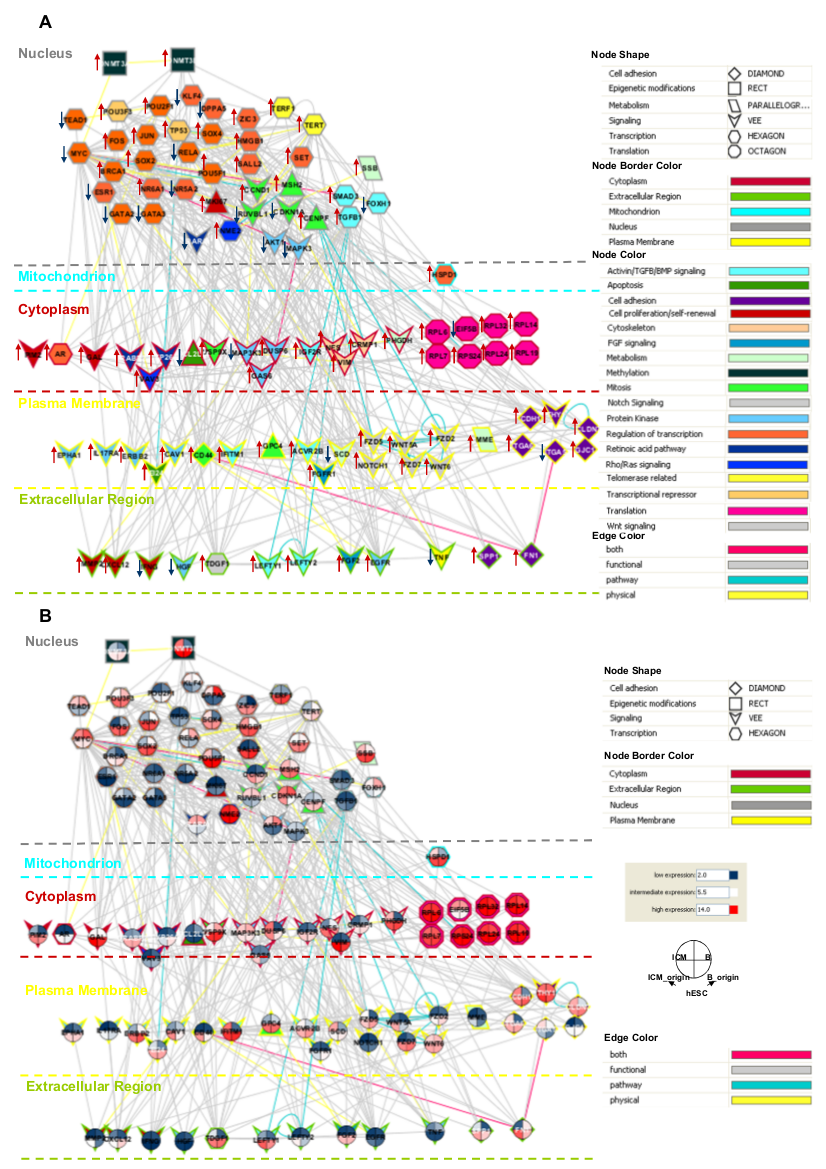

Supplement: Figure S2 — (A) Cytoscape analysis of all gene markers showing any interaction from the in vitro pluripotency signature. Node border color refers to cell localization, node shape to general function, and node color to specific function in the cell. Edge color refers to physical interactions, biochemical interactions or to both; when not specified functional interaction is assumed. Upstream arrow (red) means up-regulation versus single blastomeres and ICM, and downstream arrow (blue) means down-regulation versus blastomeres and ICMs. (B) Microarray data value representations of the in vitro pluripotency signature markers showing any interactions. (TIFF) [file pone.0062135.s002.tiff]

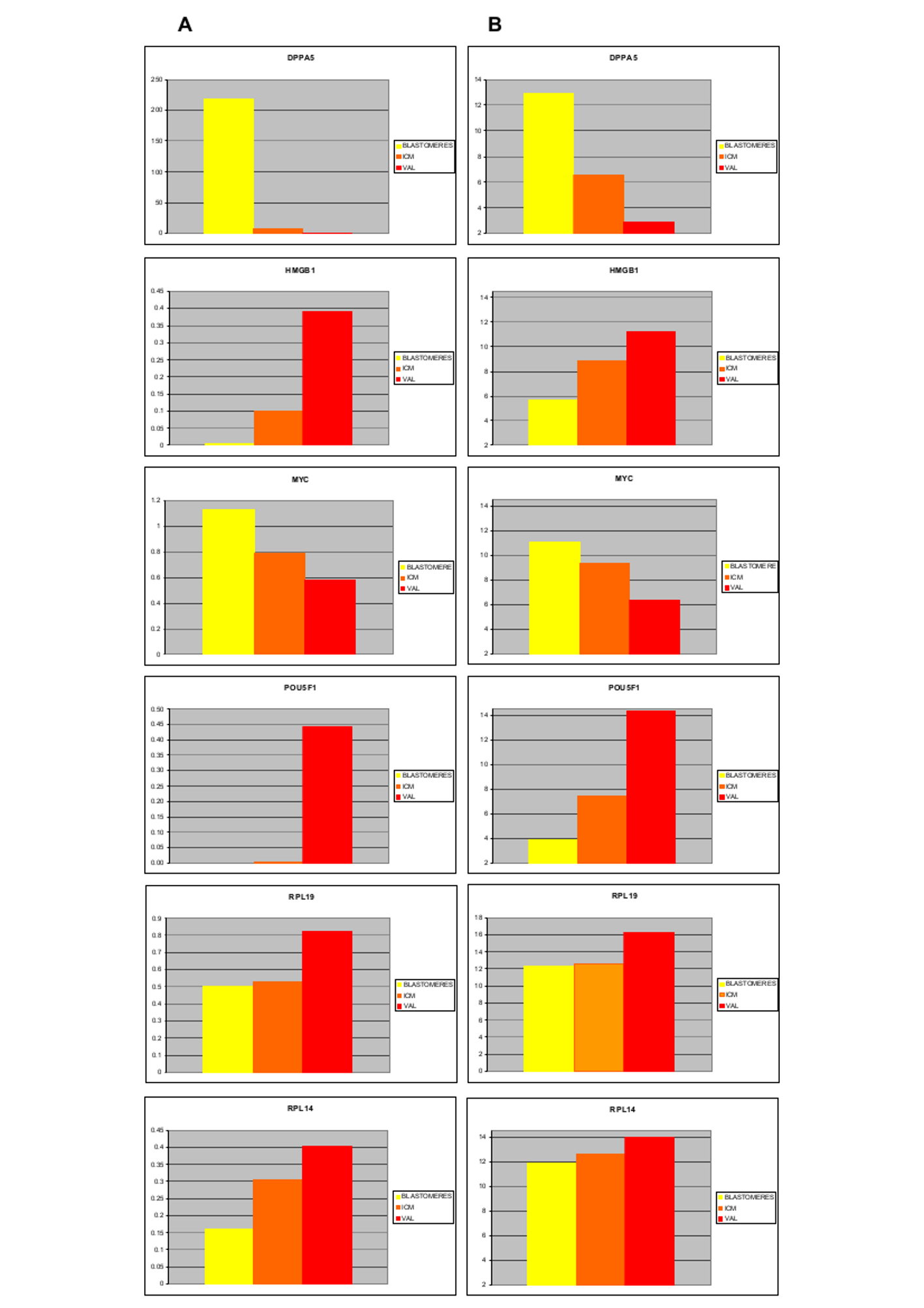

Supplement: Figure S3 — Validation of microarray results by real-time quantitative PCR. (A) Results obtained from qPCR analysis performed on non-amplified blastomeres, ICMs, and hESCs (VAL-5,-7, -8, 10B, -11B) for DDPA5, HMGB1, MYC, POU5F1, RPL14 and RPL19. RPS24 were used as references. (B) Microarray data corresponding to genes analyzed. (TIFF) [file pone.0062135.s003.tiff]
